# Editorial: Dietary and lifestyle factors associated with hypertension among children and adolescents

**DOI:** 10.3389/fped.2023.1212385

**Published:** 2023-06-14

**Authors:** Jia Hu, Yi-De Yang

**Affiliations:** ^1^ Department of School Health, Suzhou Center for Disease Prevention and Control, Suzhou, China; ^2^ State Key Laboratory of Reproductive Medicine, School of Public Health, Nanjing Medical University, Nanjing, China; ^3^ Suzhou Institute of Advanced Study in Public Health, Gusu School, Nanjing Medical University, Suzhou, China; ^4^ Key Laboratory of Molecular Epidemiology of Hunan Province, School of Medicine, Hunan Normal University, Changsha, China

**Keywords:** hypertension, healthy lifestyle, growth and development, obesity, dietary pattern

**Editorial on the Research Topic** Dietary and lifestyle factors associated with hypertension among children and adolescents

Hypertension (HTN) is a prevalent chronic medical condition characterized by an increased force of blood pushing against the walls of arteries. It is also considered one of the complex diseases with high complications and lethality in cardiovascular diseases (CVD) (1). This disease is usually diagnosed in adults, but the onset of HTN is gradually becoming younger as lifestyle and dietary patterns change and has become a current major public health problem (2). Notably, the short-term hazards of HTN in childhood and adolescence are related to target organ damage, while its long-term effects include structural and functional changes in vital organs such as the heart, kidneys, eyes, and brain (3). Furthermore, it increases the risk of developing CVD later in life. For example, a study conducted on children and adolescents diagnosed with early HTN revealed that around 51% of the subjects had left atrial dimension >95% upper confidence limit of normal children (4). Hence, early identification of HTN risk factors and advancing the window for prevention and treatment in childhood and adolescence hold significant clinical and public health implications. A total of four papers published under this research topic presents compelling evidence on dietary and lifestyle factors linked with diverse blood pressure outcomes.

Compared to adults, blood pressure standards for children and adolescents are predominantly determined by sex, age, and height (5, 6). Generally, elevated blood pressure (EBP) is defined as high systolic and/or diastolic blood pressure at a single visit, while HTN is diagnosed as EBP at three separate consecutive visits (each measured at an interval of no less than 1–2 weeks) (5, 6). Studies indicate that around 70%–80% of individuals diagnosed with EBP at the first visit are subsequently excluded as having HTN by the third visit. However, most previous studies have focused only on EBP in children and adolescents at a single visit, with EBP prevalence ranging from 2.2% to 26.4%. It may seriously overestimate the prevalence of HTN in children and adolescents (7–9). Interestingly, Hu et al. applied distinct blood pressure criteria to investigate the prevalence of HTN and its risk factors in children and adolescents at three separate visits. According to the 2018 Chinese health standard, this study discovered that HTN prevalence at three separate visits were 20.4%, 6.3%, and 3.1%, respectively, which were lower than the results of the American Association of Pediatrics standard and 2018 Chinese guidelines for prevention and treatment of HTN. Boys, older age, urban residents, high-socioeconomic status, and body mass index category including overweight and obesity were associated with confirmed HTN, and early and effective intervention around risk factors for HTN should be taken. This study provides valuable insights into the prevalence of HTN among children and adolescents in China and its related risk factors, which can drive relevant public health policies and interventions implementation.

One of the main causes of HTN is an unhealthy diet and lifestyle. Many studies have demonstrated the relationship between blood pressure levels in children and adolescents and dietary and lifestyle factors including excessive salt intake, high fat intake, obesity, physical inactivity, sedentary behavior, and insufficient sleep duration (10). These factors may also contribute to other metabolic disorders, such as insulin resistance, dyslipidemia, and inflammation, which can further aggravate HTN and its consequences (11, 12). In a cross-sectional study of 5,546 children aged 6–17 years, Li et al. investigated the association between changes in weight status from birth to childhood and EBP in childhood. Children who had incident or persistently high weight from birth to childhood had a greater risk of developing EBP in childhood, but those who had high birth weight but changed to normal weight in youth did not have an elevated risk, which is similar to the results of studies of weight change from childhood to adulthood (13). As we know, there is a growing worldwide trend for young people to delay marriage and childbirth. Within the current topic, Deng et al. found that children born to younger and older parents were at higher risk for EBP compared to middle-aged parents. Interestingly, the adverse effects of this could be corrected by developing a healthy lifestyle (ideal BMI, physical activity, sleep duration, and eating behavior) during childhood and adolescence. This study has important implications for the Chinese government's implementation of a three-child policy. Additionally, a study conducted by Dai et al. including 105 Chinese university students revealed that nap duration was independently associated with 24-h ambulatory blood pressure variability. Especially, individuals with more than 60 min of daytime napping had significantly higher BPV than those without daytime napping. These studies highlight the possibility that maintaining a healthy lifestyle and diet can reduce or prevent the risk of EBP.

In conclusion, HTN is a prevalent and modifiable risk factor for CVD, which can have its origins in childhood. Dietary and lifestyle factors are crucial in the development and progression of HTN among pediatric populations. Unfortunately, most studies on this topic are cross-sectional, and future research should further clarify their causal associations with pediatric HTN. We hope that future research will demonstrate how adopting a healthy diet and lifestyle from an early age can benefit for the health children and adolescents or even health in adulthood.

## References

[1] Collaborators GBDRF. Global, regional, and national comparative risk assessment of 79 behavioural, environmental and occupational, and metabolic risks or clusters of risks, 1990-2015: a systematic analysis for the global burden of disease study 2015. Lancet. (2016) 388(10053):1659–724. 10.1016/S0140-6736(16)31679-8 27733284 PMC5388856

[2] Roth GA Abate D Abate KH Abay SM Abbafati C Abbasi N Global, regional, and national age-sex-specific mortality for 282 causes of death in 195 countries and territories, 1980–2017: a systematic analysis for the global burden of disease study 2017. Lancet. (2018) 392(10159):1736–88. 10.1016/S0140-6736(18)32203-7 30496103 PMC6227606

[3] Daniels SR Pratt CA Hayman LL. Reduction of risk for cardiovascular disease in children and adolescents. Circulation. (2011) 124(15):1673–86. 10.1161/CIRCULATIONAHA.110.016170 21986774 PMC3751579

[4] Daniels SR Witt SA Glascock B Khoury PR Kimball TR. Left atrial size in children with hypertension: the influence of obesity, blood pressure, and left ventricular mass. J Pediatr. (2002) 141(2):186–90. 10.1067/mpd.2002.125851 12183712

[5] Flynn JT Kaelber DC Baker-Smith CM Blowey D Carroll AE Daniels SR Clinical practice guideline for screening and management of high blood pressure in children and adolescents. Pediatrics. (2017) 140(3):e20171904. 10.1542/peds.2017-1904 28827377

[6] Dong Y Ma J Song Y Dong B Wang Z Yang Z National blood pressure reference for Chinese han children and adolescents aged 7 to 17 years. Hypertension. (2017) 70(5):897–906. 10.1161/HYPERTENSIONAHA.117.09983 28923902 PMC5722224

[7] Teng H Hu J Ge W Dai Q Liu J Xiao C Body mass index trajectories during 6-18 years old and the risk of hypertension in young adult: a longitudinal study in Chinese population. Int J Hypertens. (2021) 2021:6646868. 10.1155/2021/6646868 34327015 PMC8302370

[8] Wang X Hu J Huang S Yang Z Dong Y Dong B Exploring overweight risk trajectories during childhood and their associations with elevated blood pressure at late adolescence: a retrospective cohort study. Hypertension. (2022) 79(8):1605–13. 10.1161/HYPERTENSIONAHA.121.18714 35094521

[9] Sun J Steffen LM Ma C Liang Y Xi B. Definition of pediatric hypertension: are blood pressure measurements on three separate occasions necessary? Hypertens Res. (2017) 40(5):496–503. 10.1038/hr.2016.179 28077857

[10] Couch SC Saelens BE Khoury PR Dart KB Hinn K Mitsnefes MM Dietary approaches to stop hypertension dietary intervention improves blood pressure and vascular health in youth with elevated blood pressure. Hypertension. (2021) 77(1):241–51. 10.1161/HYPERTENSIONAHA.120.16156 33190559 PMC7725858

[11] Deng Y Huang C Su J Pan CW Ke C. Identification of biomarkers for essential hypertension based on metabolomics. Nutr Metab Cardiovasc Dis. (2021) 31(2):382–95. 10.1016/j.numecd.2020.11.023 33495028

[12] Oparil S Acelajado MC Bakris GL Berlowitz DR Cifkova R Dominiczak AF Hypertension. Nat Rev Dis Primers. (2018) 4:18014. 10.1038/nrdp.2018.14 29565029 PMC6477925

[13] Sun J Xi B Yang L Zhao M Juonala M Magnussen CG. Weight change from childhood to adulthood and cardiovascular risk factors and outcomes in adulthood: a systematic review of the literature. Obes Rev. (2021) 22(3):e13138. 10.1111/obr.13138 32875696

